# Chondromalacia patella increases the risk of herpes zoster: a population-based study

**DOI:** 10.1186/s12891-022-05929-y

**Published:** 2022-11-08

**Authors:** Chia-Hung Chen, Yung-Chi Cheng, Hsin-Yi Yang, Ching-Fang Tsai, Chao-Yu Hsu, Der-Shin Ke, Wen-Che Hsieh

**Affiliations:** 1 Department of Medical Education, Ditmanson Medical Foundation, Chia-Yi Christian Hospital, Chia-Yi, Taiwan; 2 Department of Medical Imaging, Ditmanson Medical Foundation, Chia-Yi Christian Hospital, Chia-Yi, Taiwan; 3 Department of Rehabilitation, Ditmanson Medical Foundation, Chia-Yi Christian Hospital, Chia-Yi, Taiwan; 4 Clinical Data Center, Department of Medical Research, Ditmanson Medical Foundation, Chia-Yi Christian Hospital, Chia-Yi, Taiwan; 5 Clinical Medicine Research Center, Department of Medical Research, Ditmanson Medical Foundation, Chia-Yi Christian Hospital, Chia-Yi, Taiwan; 6 Department of Optometry, Central Taiwan University of Science and Technology, Taichung, Taiwan; 7 Department of Medical Imaging and Radiological Sciences, Central Taiwan University of Science and Technology, Taichung, Taiwan; 8 Center for General Education, National Taichung University of Science and Technology, Taichung, Taiwan; 9 Department of General Education, National Chin-Yi University of Technology, Taichung, Taiwan; 10 Department of Neurology, Ditmanson Medical Foundation, Chia-Yi Christian Hospital, No 539 Zhongxia Road, Chia-Yi, Taiwan; 11 Department of Chinese Medicine, Ditmanson Medical Foundation, Chia-Yi Christian Hospital, No 539 Zhongxia Road, Chia-Yi, Taiwan

**Keywords:** Chondromalacia patella, Herpes zoster, Women

## Abstract

**Background:**

The reactivation of herpes zoster (HZ) is associated with disease stress. However, the relationship between chondromalacia patella (CMP) and HZ remains poorly understood. This study investigated the relationship between CMP and the risk of developing HZ.

**Methods:**

Data were collected from the Taiwan’s National Health Insurance Research Database. Patients with CMP diagnosed between 2000 and 2017 were assigned to the case group; patients without CMP were randomly selected from the same database and paired with controls matched by age and sex. The primary outcome was a diagnosis of HZ. All patients were followed until their diagnosis of HZ, their withdrawal from the NHI program, their death, or the end of 2017, whichever was earliest. The risk of developing HZ was compared between the case and control groups.

**Results:**

In total, 22,710 patients with CMP and 90,840 matched controls were enrolled. The overall incidence rates of HZ in the CMP and control cohorts were 7.94 and 7.35 per 1,000 person-years, respectively. After potential confounders were controlled for, the case group exhibited a higher risk of HZ than did the control group [adjusted hazard ratio (aHR) = 1.06, p < 0.05]. In a stratification analysis by age, patients over 65 years old in the CMP group exhibited a higher risk of HZ than did those in the control group (aHR = 1.22, p < 0.01). In a stratification analysis by sex, women with CMP were at greater risk of developing HZ than women without CMP (aHR = 1.18, p < 0.01).

**Conclusion:**

Patients with CMP, especially elder adults and women, exhibited a higher risk of HZ. The HZ risk of patients with CMP should thus be assessed, and the necessity of HZ vaccination should be informed.

## Background

Chondromalacia patella (CMP) involves degenerative changes in cartilage due to a poor alignment of the kneecap, and is a highly common cause of chronic knee pain. In a postmortem study involving 59 persons, cartilaginous change was discovered in 91 of the individuals’ 118 patellae [1]. In a study by Özdemir and Kavak involving military recruits with anterior knee pain, magnetic resonance imaging examinations revealed that 58.7% of the recruits had CMP [2]. Perineural injection plus physical therapy is an effective conservative management strategy to relieve pain and stiffness [3].

Herpes zoster (HZ) is a skin disease caused by the reactivation of the varicella zoster virus (VZV). The prevalence of HZ is 18.54 per 1,000 persons [4], and the incidence of HZ increased every year. In a population-based study, Kawai et al. reported that the incidence rates of HZ were 0.76 and 3.15 per 1000 person-years from 1945 to 1949 and from 2000 to 2007, respectively [5]. Thompson et al. reported that in the United States, the incidence of HZ increased from 286.0 to 579.6 per 100,000 person-years from 1994 to 2018, which is an average annual increase of 3.1% [6]. The incidence of post-herpetic neuralgia, a painful complication of HZ, was reported to be 57.5 per 100,000 person-years [6]. Several procedures, including steroid injection and radiofrequency ablation, may be effective for treating post-herpetic neuralgia [7].

CMP is characterized by chronic knee pain, and chronic pain is significantly associated with depression [8]. Rapti et al. reported that 22.5% of their participants with chronic pain had depression, as evaluated using the Patient Health Questionnaire*-*9 [9]. Chronic pain-related conditions, such as fatigue and frailty, also increase depression risk [10–13]. Thus, because depression increases HZ risk [14], the patients with chronic pain may have a higher risk to have HZ development. Furthermore, chronic pain-related diseases including chronic interstitial cystitis [15], varicocele [16] and endometriosis [17] in the urogenital system and also adhesive capsulitis of the shoulder [18], sciatica [19], lateral epicondylitis [20], plantar fascial fibromatosis [21], and de Quervain syndrome [22] in the musculoskeletal system are strongly associated with HZ development. Therefore, patients with CMP are also likely to exhibit an increased risk of HZ. In this study, we investigated the risk of HZ reactivation in patients with CMP.

## Materials and methods

### Data source

The National Health Insurance (NHI) program, which employs a government-run single-payer model, has been in operation since 1995. More than 99% of Taiwanese people are enrolled in the program. All medical claims of enrolled residents are recorded in the NHI Research Database (NHIRD). In this study, we analyzed the 2000 Longitudinal Generation Tracking Database (LGTD2000), which is a subset of the NHIRD, and contains the records of 2 million insured individuals randomly selected from the NHIRD. The information of each patient enrolled in this study, including records of clinic visits, hospitalization and medication was well documented in the NHIRD. To protect the patient’s privacy, the patients’ personally identifiable information is encrypted in all NHI data. The diagnostic codes of CMP, HZ and all comorbidities were defined in accordance with the International Classification of Diseases, Ninth Revision, Clinical Modification (ICD-9-CM) between 2000 and 2015, and the International Classification of Diseases, Tenth Revision, Clinical Modification (ICD-10-CM) after 2015. This study was approved by the institutional review board of Chia-Yi Christian Hospital, in Chia-Yi, Taiwan (IRB2020113). All methods were carried out in accordance with declaration of Helsinki in our manuscript.

### Study population

Patients with an initial diagnosis of CMP made between 2000 and 2017, (ICD-9-CM: 717.7; ICD-10-CM: M22.4) were enrolled in the case cohort. Patients with a previous history of HZ or who were younger than 20 years were excluded from the study. The patients in the control group were matched by age and sex with the patients in the case group at a 4:1 ratio. The index date was defined as the date of initial diagnosis of CMP in the case cohort. All the patients were followed until their diagnosis of HZ, their withdrawal from the NHI program, their death, or the end of 2017, whichever was earliest.

### Main outcome and relevant variables

The primary outcome of this study was a HZ diagnosis (ICD-9-CM: 053; ICD-10-CM: B02). Several diseases, namely diabetes (ICD-9-CM: 250; ICD-10-CM: E08-E13), chronic kidney disease (CKD; ICD-9-CM: 585, ICD-10-CM: N18), coronary artery disease (CAD; ICD-9-CM: 410–414; ICD-10-CM: I20-I22, I24, I25), depression (ICD-9-CM: 296.2, 296.3, 300.4, 311; ICD-10-CM: F32, F33, F34.1), and cancer (ICD-9-CM: 140–208; ICD-10-CM: C00-C26, C30-C34, C37-C41, C43-C50, C53-C55, C4A, C7A, D03, Z51.12), were considered as comorbidities in this study.

### Statistical analysis

The distributions of the patients’ characteristics and comorbidities between the CMP and control cohorts were compared. A chi-square test and a *t*-test were used to analyze categorical and continuous variables. The hazard ratios and 95% confidence intervals (CIs) were estimated using a Cox proportional hazards model. Multivariate analysis was conducted after age, sex and comorbidities were adjusted for. The Kaplan-Meier method was used to assess the cumulative incidence of HZ, and the differences between groups were evaluated using a log-rank test. A two-tailed *p* value of < 0.05 was considered statistically significant. All data were analyzed using SAS 9.4 for Windows (SAS Institute, Cary, NC, USA).

## Results

**Fig. 1:**
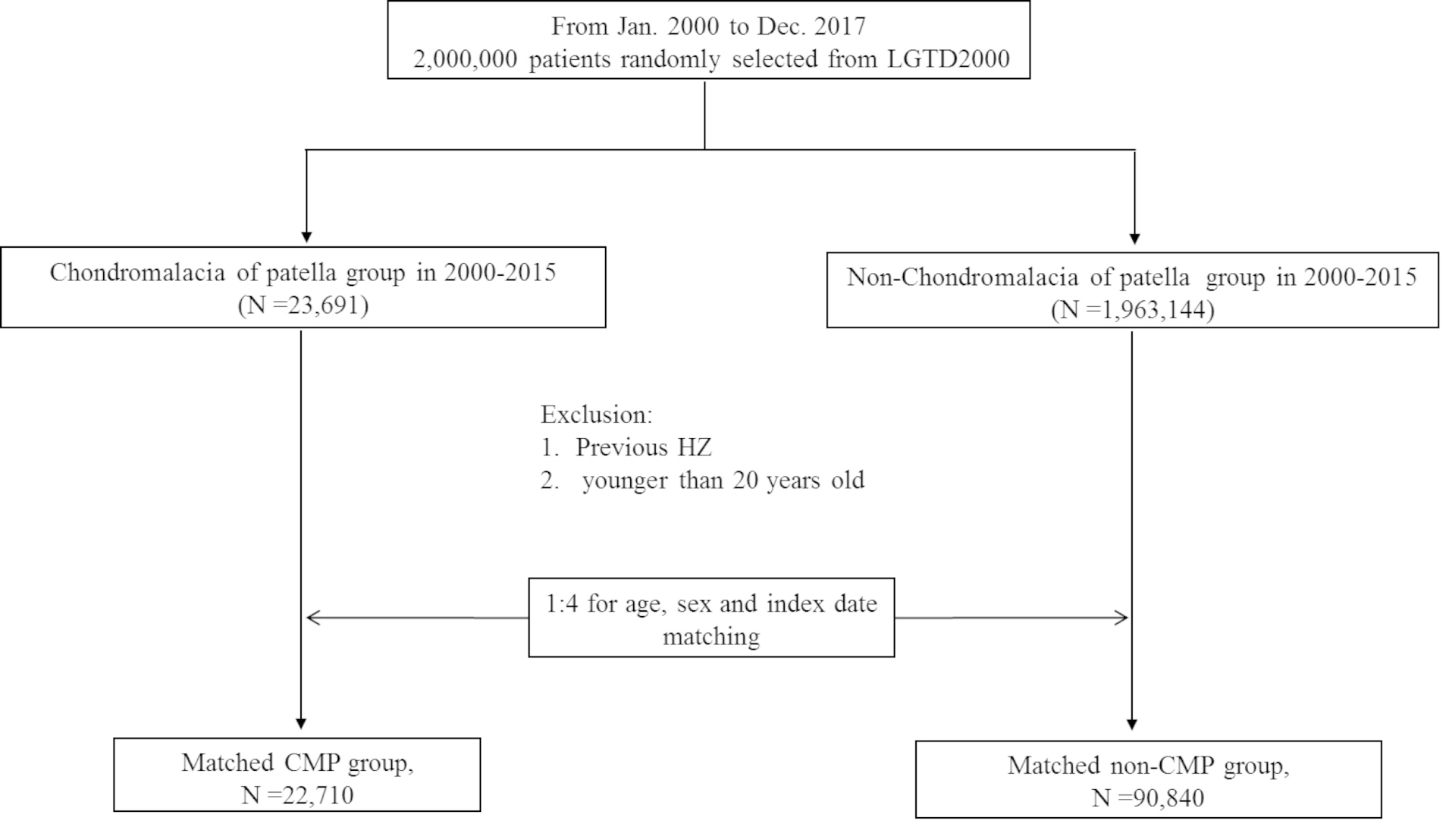
Flowchart for data collection process

**Fig. 2:**
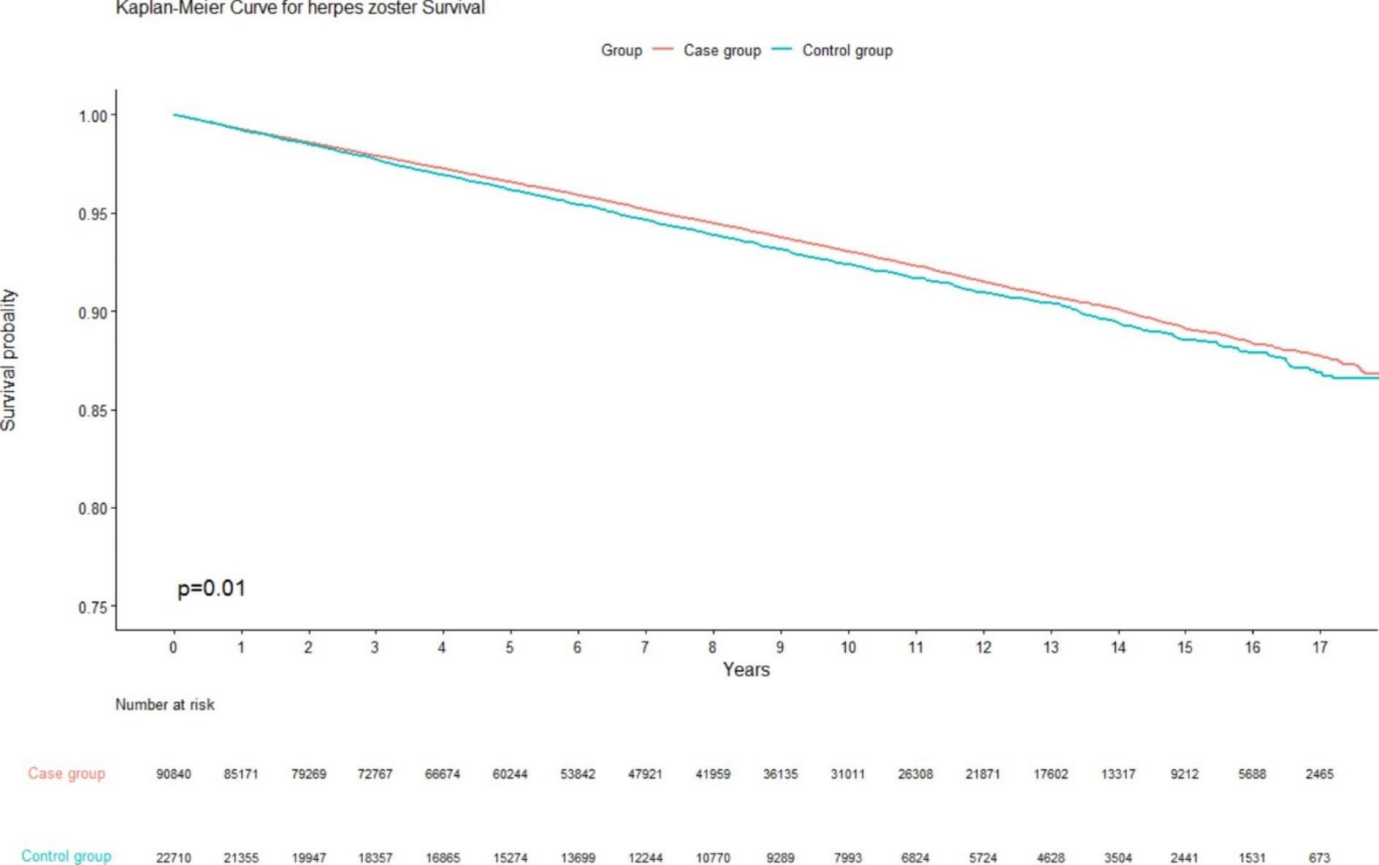
The cumulative incidence of HZ in the patients with CMP was significantly higher than the patients without CMP.

A total of 22,710 patients with CMP and 90,840 patients without CMP were enrolled in the final study (Fig. 1). The mean follow-up period was 7.88 ± 4.88 years for control group, and 8.01 ± 4.91 years for case group. The patients’ characteristics and comorbidities are listed in Table 1. After matching, the age and sex distributions of the cohorts were similar. The patients with CMP exhibited higher rates of CAD and depression than did the patients without CMP. The cumulative incidence of HZ among the patients with CMP was significantly higher than that among the patients without CMP (Fig. 2).

**Table 1:** Demographic characteristics and comorbidities in the patients with and without chondromalacia of patella

	Chondromalacia of patella		p-value
	**No**	**Yes**	
**Variable**	**N = 90,840**	** N = 22,710**	
Age			
≤ 49	61,128 (67.29)	15,282 (67.29)	1.000
50–64	21,144 (23.28)	5286 (23.28)	
65+	8568 (9.43)	2142 (9.43)	
Mean ± SD^&^	41.69 ± 16.37	41.69 ± 16.37	1.000
Sex			
Female	55,092 (60.65)	13,773 (60.65)	1.000
Male	35,748 (39.35)	8937 (39.35)	
Comorbidity			
Diabetes	7429 (8.18)	1995 (8.78)	0.003
Chronic Kidney Disease	1053 (1.16)	265 (1.17)	0.923
Coronary Artery Disease	5938 (6.54)	1920 (8.45)	< 0.001
Depression	4233 (4.66)	1598 (7.04)	< 0.001
Cancer	2759 (3.04)	719 (3.17)	0.314

Chi-square test; &: t-test

The incidence of HZ was higher among the patients with CMP than among the patients without CMP (7.94 and 7.35 per 1000 person-years, respectively) (Table 2). The patients with CMP were 1.06 times more likely to develop HZ than were those without CMP. HZ risk increased with age: compared with patients aged ≤ 49 years, patients aged 50–64 and > 65 years were 2.41 (95% CI = 2.28, 2.55) and 2.69 (95% CI = 2.49, 2.91) times respectively, as likely to develop HZ.

**Table 2:** The incidence and risk factors of herpes zoster

Variables		Event		PY	Rate^#^ (per 1000 PY)	Crude HR	Multivariate HR^&^
						**(95% CI)**	**(95% CI)**
Chondromalacia of patella							
No	5265		716251.9		7.35	1.00	1.00
Yes	1448		182321.0		7.94	1.08 (1.02–1.14)**	1.06 (1.00-1.13)*
Age							
≤ 49	3249		644208.3		5.04	1.00	1.00
50–64	2442		187042.5		13.06	2.63 (2.50–2.77)***	2.41 (2.28–2.55)***
65+	1022		67322.1		15.18	3.10 (2.88–3.32)***	2.69 (2.49–2.91)***
Sex							
Female	4663		548359.4		8.50	1.00	1.00
Male	2050		350213.5		5.85	0.69 (0.66–0.73)***	0.81 (0.77–0.85)***
Diabetes							
No	5950		839308.0		7.09	1.00	1.00
Yes	763		59264.9		12.87	1.84 (1.71–1.99)***	1.07 (0.99–1.16)
CKD							
No	6615		891938.0		7.42	1.00	1.00
Yes	98		6634.9		14.77	2.03 (1.66–2.48)***	1.19 (0.97–1.46)
CAD							
No	5980		848569.0		7.05	1.00	1.00
Yes	733		50003.9		14.66	2.12 (1.96–2.29)***	1.20 (1.10–1.30)***
Depression							
No	6316		862744.4		7.32	1.00	1.00
Yes	397		35828.5		11.08	1.54 (1.39–1.70)***	1.18 (1.07–1.31)**
Cancer							
No	6398		879227.5		7.28	1.00	1.00
Yes	315		19345.4		16.28	2.28 (2.03–2.55)***	1.50 (1.34–1.68)***

Rate^#^, incidence rate, per 1,000 person-years; Crude HR, relative hazard ratio; Adjusted HR^&^: multivariable analysis including age, sex, and comorbidities of diabetes, CKD, CAD, depression and cancer. *p < 0.05, **p < 0.01, ***p < 0.001. CKD: chronic kidney disease; CAD: coronary artery disease

The effects of CMP on the development of HZ as related to age, sex and the presence of comorbidities are described in Table 3. The patients aged 50–64 and > 65 years, with CMP were 1.10 (95% CI = 1.00, 1.21) and 1.22 (95% CI = 1.06, 1.41) times respectively, more likely to develop HZ compared with patients without CMP in their respective age groups. Women with CMP were 1.18 times (95% CI = 1.07, 1.31) more likely to develop HZ than were women without CMP. No significant difference in HZ risk was identified between the patients with CMP with or without comorbidities and the patients without CMP. However, the patients with CMP, whether with or without comorbidities, still exhibited a slightly higher incidence of HZ than did those without CMP (7.80 vs. 6.44 per 1,000 person-years among patients with no comorbidities; 13.09 vs. 12.81 per 1,000 person-years among patients with comorbidities) (Table 3).

**Table 3:** Incidence of herpes zoster by age, sex and comorbidities, and Cox model measured hazards ratio for patients with chondromalacia of patella

Variables	Chondromalacia of patella No Yes						Crude HR*	Multivariate HR^&^
	**Event**	**PY**	**Rate** ^**#**^	**Event**	**PY**	**Rate** ^**#**^	**(95% CI)**	**(95% CI)**
Age								
≤ 49	2595	513934.4	5.05	654	130273.8	5.02	0.99 (0.91–1.08)	0.98 (0.90–1.06)
50–64	1901	149089.8	12.75	541	37952.7	14.25	1.12 (1.02–1.23)*	1.10 (1.00-1.21)*
65+	769	53227.6	14.45	253	14094.5	17.95	1.24 (1.08–1.43)**	1.22 (1.06–1.41)**
Sex								
Female	3693	436871.9	8.45	970	111487.5	8.70	1.20 (1.08–1.33)***	1.18 (1.07–1.31)**
Male	1572	279380.0	5.63	478	70833.6	6.75	1.03 (0.96–1.10)	1.01 (0.94–1.08)
Comorbidity^§^								
No	3950	613587.1	6.44	1016	149318.1	6.80	1.06 (0.99–1.13)	1.06 (0.99–1.14)
Yes	1315	102664.7	12.81	432	33002.9	13.09	1.02 (0.91–1.14)	1.05 (0.94–1.17)

Rate^#^, incidence rate, per 1,000 person-years; Crude HR*, relative hazard ratio; Adjusted HR^&^: multivariable analysis including age, sex, and comorbidities^§^. Patients with any comorbidities of diabetes, CKD, CAD, depression, and cancer were classified into the comorbidity group. *p < 0.05, **p < 0.01, ***p < 0.001

The Schoenfeld residuals are calculated for each regression variable to see if each variable independently satisfies the assumptions of the Cox model (Table 4). We used Stratified Cox regression to check, the case group still has a higher risk (Table 5).

**Table 4:** Cox proportional-hazards model assumption test

	chisq	df	p
Group	1.86	1	0.172
Sex	9.67	1	0.002
Age	1.97	1	0.160
Diabetes	0.28	1	0.596
CAD	0.23	1	0.628
CHD	1.55	1	0.213
Depression	0.26	1	0.608
cancer	0.22	1	0.642
Globle	14.33	8	0.074

CKD: chronic kidney disease; CAD: coronary artery disease

**Table 5:** Checking by stratified Cox regression, the case group still has a higher risk

	Adjusted HR (95% CI)	p-value
Group		
No	1.00	
Yes	1.06 (1–1.12)	0.047
Age		
≦ 49	1.00	
50–64	2.41 (2.28–2.54)	< 0.001
65+	2.69 (2.49–2.91)	< 0.001
DM		
No	1.00	
Yes	1.07 (0.99–1.16)	0.097
CKD		
No	1.00	
Yes	1.19 (0.97–1.45)	0.096
CHD		
No	1.00	
Yes	1.2 (1.1–1.3)	< 0.001
Depression		
No	1.00	
Yes	1.19 (1.07–1.32)	0.001
Cancer		
No	1.00	
Yes	1.5 (1.34–1.69)	< 0.001

## Discussion

In this study, we investigated the relationship between CMP and HZ, and discovered that patients with CMP, especially older adults and women, exhibited a significantly higher risk of developing HZ. Chronic pain is a frequently complaint of patients with CMP and is associated with several conditions, including fatigue, frailty and depression that may exacerbate the stress of patients with CMP.

Aili et al. reported that fatigue was a predictive factor for the onset of chronic pain by the 5th-year of follow-up [23]. Manning et al. reported that fatigue sensitivity was a significant predictor of the severity of chronic pain [24]. Al-Rawaf et al. used microRNAs as biomarkers of pain intensity to explore the correlation between patients’ expression of microRNAs and levels of inflammatory markers. MicroRNAs expression was significantly associated with interleukin-6, tumor necrosis factor-α, and cyclooxygenase-2 levels in patients with fatigue [25]. Fatigue may commonly be due to increased levels of pro-inflammatory cytokines such as interleukin-1, interleukin-6 and tumor necrosis factor-α. In addition, pain and behavioral symptoms might also be exacerbated by pro-inflammatory processes [26]. Thus, inflammation may serve as a mechanism underlying both pain and fatigue.

Corfield et al. reported that the incidence of depression was significantly higher among patients with fatigue than among patients without fatigue, and that fatigue was a strong predictor of depression. Moreover, the authors determined that patients with fatigue or depression are twice more likely to have both conditions than to have only one. In addition, the symptoms of fatigue and depression may overlap [10]. Both conditions are associated with an increased activation of the immune system, which affects both the central and peripheral nervous system. Lee and Giuliani postulated that an immunopsychiatric link must exist between fatigue and depression [11].

Nakai et al. reported that a strong association between chronic pain and frailty (as defined by five items: exhaustion, slowness, weakness, low physical activity, and weight loss) or pre-frailty [27]. A systematic review and meta-analysis reported that prevalence rates of frailty and pre-frailty in patients with chronic pain were 18% and 43%, respectively. The prevalence rates of chronic pain were 50% and 37% among patients with frailty and pre-frailty, respectively. Furthermore, the authors found that patients with chronic pain were a 1.85 times more likely to have frailty than those without chronic pain [28]. Otones Reyes et al. investigated the relationship between chronic pain and frailty, and discovered that 45% of patients with frailty also had chronic pain. The authors concluded that chronic pain was a predictor of frailty [29].

Soysal et al. reported a reciprocal interaction exists between depression and frailty. The prevalence of depression among the patients with frailty enrolled in their study was 38.6%, and the patients with frailty were 2.64 times as likely to develop depression. Moreover, the prevalence of frailty was 40.4% among the patients with depression, and the patients with depression were 3.72 times as likely to have frailty [12]. Oyon et al. observed a dose-response relationship between severity of depression and risk of frailty; the severity of depression exhibited a higher association with frailty than with other psychosocial factors [13]. On the basis of the aforementioned reports, frailty and depression can be concluded to be frequently concomitant conditions.

Irwinet et al. compared VZV-specific responder cell frequency (RCF) in patients with and without depression to identify the groups’ respective levels of VZV-specific cellular immunity. VZV-specific RCF was significantly lower in the patients with depression than in patients without depression; therefore, the authors concluded that the VZV-specific cellular immunity of patients with depression was significantly lower than that of patients without depression [30].

Liao et al. conducted a population-based study to evaluate the incidence of HZ among patients with depression, and reported that the incidence of HZ was higher among patients with depression than among patients without depression (4.58 and 3.54 per 1,000 person-years, respectively; aHR = 1.1). The authors also discovered that middle-aged patients exhibited the highest risk of HZ compared with any other age group [31]. Choi et al. conducted a study employing a sample more than twice the size of that employed by Liao et al. to assess the incidence of HZ among patients with depression and similarly reported that the incidence of HZ was higher among patients with depression than among patients without depression (6.8% and 6.3%, respectively). In addition, they reported that patients with depression were 1.09 times more likely to develop HZ than were patients without depression, and that middle-aged women exhibited the highest risk of developing HZ compared with other age and sex group [32].

In our study, the incidence of depression was higher among the case group than among the control group (Table 1), and according to the univariate analysis, HZ risk was higher among patients with any comorbidities than among those without comorbidities (Table 2). However, the incidence of HZ among the patients with CMP with or without comorbidities was higher than that of the patients without CMP (Table 3). CMP must be stressful for involving persons. CMP can seriously affect patient’ wellbeing, and the risk of HZ should be assessed in patients with CMP, especially women.

This study does have some limitations. First, the diagnosis codes were obtained from Taiwan’s NHIRD. CMP could be diagnosed by patient’s medical history and physical examination or imaging findings. Most patients with HZ were diagnosed by the appearance of painful herpetiform vesicles in a restricted dermatomal distribution. The patients’ diagnosis of CMP or HZ were made by different specialists; therefore, diagnosis bias may be existed. However, Taiwan’s NHI Administration (NHIA) has a strict review system. When a prescription or results of an examination are returned to the NHIA with a diagnosis, the diagnosis is reviewed under strict protocols. Any impropriety in diagnosis, drug prescription or examination is punishable by law. Therefore, although diagnosis codes were issued by different specialists, diagnoses analyzed in this study were still generally accurate. Second, the severity of a disease affects its subsequent treatment and results in different prognoses. The ICD-10-CM has been officially adopted since 2016; however, because the diagnostic codes used in this study are mainly ICD-9-CM codes, the severity of each patient’s disease cannot be distinguished. Third, NHIRD data do not fully cover the life-style of the patients. Each patient’s dietary (vegetarian or non-vegetarian), smoking (number of cigarettes), drinking (beer or spirits), and exercise (number of exercises and length of time) habits may affect the appearance, severity and prognosis of their disease. Fourth, self-financed treatments are not consistently recorded in the NHIRD. Few patients with CMP who experience chronic pain may seek alternative treatments, such as herbal medicine, acupuncture or therapeutic massage. These self-financed treatments may not be recorded in the NHIRD, but may not affect the overall incidence of CMP.

Due to the unique insurance system with high accessibility, most Taiwanese will seek medical care whenever having any discomfort. Therefore, the diagnosis of HZ in this study is highly trustable and will not be affected by presence or not of CMP. This is a retrospective cohort study, the temporality between diagnosis of CMP and occurrence of HZ is preserved, therefore possibility of a causal association is present. Despite the limitations of this study, our finding of a strong positive correlation between CMP and HZ from analysis of NHIRD data, contributes to the literature and clinical practice. Moreover, the results can provide a reference for population medicine research in the future.

## Conclusion

In this study, the patients with CMP, especially older adults and women, exhibited a significantly higher risk of HZ reactivation. The risk of HZ should be carefully assessed in patients with CMP, and the necessity of HZ vaccination should be informed.

## Data Availability

Data are available from the National Health Insurance Research Database which provided by the Ministry of Health and Welfare (MOHW), Taiwan. However, data cannot be disclosed because of data protection laws. Data requests should be applied following a formal procedure (http://dep.mohw.gov.tw). Please contact the staff of MOHW (Email: stdlwu@mohw.gov.tw) for assistance.

## Abbreviations

CAD: Coronary artery disease
CKD: Chronic kidney disease
CMP: Chondromalacia patella
RCF: Responder cell frequency
HZ: Herpes zoster
ICD-9-CM: International classification of diseases, ninth revision, clinical modification
ICD-10-CM: International classification of diseases, tenth revision, clinical modification
LGTD: Longitudinal generation tracking database
NHI: National health insurance
NHIA: National health insurance administration
NHIRD: National health insurance research database
VZV: Varicella zoster virus
